# Homoeologous Recombination of the *V1r1*-*V1r2* Gene Cluster of Pheromone Receptors in an Allotetraploid Lineage of Teleosts

**DOI:** 10.3390/genes8110334

**Published:** 2017-11-21

**Authors:** Lei Zhong, Weimin Wang

**Affiliations:** Key Lab of Agricultural Animal Genetics, Breeding and Reproduction of Ministry of Education/Key Lab of Freshwater Animal Breeding of Ministry of Agriculture/Freshwater Aquaculture Collaborative Innovation Center of Hubei Province, College of Fisheries, Huazhong Agricultural University, Wuhan 430070, China; leiclock@mail.hzau.edu.cn

**Keywords:** homoeologous recombination, V1R, allotetraploid, directional selection, teleost

## Abstract

In contrast to other olfactory receptor families that exhibit frequent lineage-specific expansions, the vomeronasal type 1 receptor (V1R) family exhibits a canonical six-member repertoire in teleosts. *V1r1* and *V1r2* are present in no more than one copy in all examined teleosts, including salmons, which are ancient polyploids, implying strict evolutionary constraints. However, recent polyploids have not been examined. Here, we identified a young allotetraploid lineage of weatherfishes and investigated their *V1r1*-*V1r2* cluster. We found a novel pattern that the parental *V1r1*-*V1r2* clusters had recombined in the tetraploid genome and that the recombinant was nearly fixed in the tetraploid population. Subsequent analyses suggested strong selective pressure, for both a new combination of paralogs and homogeneity among gene duplicates, acting on the *V1r1*-*V1r2* pair.

## 1. Introduction

Olfaction is central to pheromone detection. Vomeronasal type 1 receptors (V1Rs), which constitute an olfactory receptor family encoding seven transmembrane G-protein-coupled receptors (GPCRs), function as pheromone receptors in vertebrates [1,2]. For example, in zebrafish, V1R2 (synonymous with ORA1 [3,4]) is a receptor for a putative sex pheromone [5]. Unlike other olfactory receptor families (odorant receptors, trace amine-associated receptors, vomeronasal type 2 receptors), which often include over ten members and exhibit lineage-specific expansions, the V1R family presents a canonical six-member repertoire in teleosts, with the exception of occasional duplications of *V1r3* and *V1r5* in some lineages [3,4,6,7]. The pairwise-arranged *V1r1* and *V1r2* are tightly linked, being oriented head-to-head, and have not been duplicated among the surveyed teleost species, including Atlantic salmon, which belongs to an order that has undergone order-specific polyploidization [6,8]. We hypothesized that *V1r1* and *V1r2* of teleosts evolved under strict evolutionary constraints selecting against heterogeneity and thus preventing divergence and preservation of duplicates. Because genes are duplicated via polyploidization (genome duplication) and duplicated genes can survive over long time scales [9,10,11], the evolutionary pattern of *V1r1* and *V1r2* in polyploid teleosts will be informative to test the hypothesis. However, little is known about *V1r1* and *V1r2* in polyploid teleosts other than Atlantic salmon, especially in recently-formed polyploid species.

Weatherfishes, from the genera *Misgurnus* and *Paramisgurnus*, are a group of small benthic loaches living in rivers, lakes, ponds and paddy fields that are distributed across East Asia and Central Europe [12]. The genus *Misgurnus* is composed of several nominal species, among which *Misgurnus anguillicaudatus* occurs primarily in southern East Asia and is regarded as a species complex because it consists of both diploid and tetraploid populations, which are morphologically indistinguishable from each other [13]. *Misgurnus bipartitus* is distributed in northern East Asia and exhibits a largely parapatric distribution pattern with *M*. *anguillicaudatus* currently. Populations of *Paramisgurnus dabryanus*, the only species of the genus *Paramisgurnus*, live sympatrically with *M. anguillicaudatus* and *M. bipartitus* [12,14].

In the present study, we investigated the composition and evolution of *V1r1* and *V1r2* in a tetraploid lineage of weather loach identified in this study, a recently-formed polyploid teleost, in comparison with that in its relative species. Here, we present our findings regarding the duplication of the *V1r1*-*V1r2* pair derived via allopolyploidization and its evolution in this young polyploid genome.

## 2. Materials and Methods

### 2.1. Data Collection

The sample collection and experiments were conducted in accordance with the national legislation of China and approved by the ethics committee of Huazhong Agricultural University, Study Number HZAUFI-2016-006.

#### 2.1.1. Sample Collection and Evaluation of Polyploidy

Fish samples were collected from four localities in China (Figure S1): Yueyang, Luoning, Daqing and Fuyuan, from south to north. Taxonomic assignment of the samples was based on morphological characteristics according to Cheng [12]. The sexes of individual loaches were determined based on sexual dimorphism, as mature males exhibit a bony plate in each pectoral fin [15]. Because there are both diploid and polyploid populations of *M. anguillicaudatus* [13], flow cytometry analysis using erythrocytes was performed to determine the ploidy status of the samples according to Yu et al. [13]. The loaches were preserved in 95% ethanol after being anaesthetized in a solution of 200 mg/L tricaine methanesulfonate (MS-222; Sigma-Aldrich, Saint Louis, MO, USA). Seven *M. anguillicaudatus* diploids from Yueyang, thirteen *M. anguillicaudatus* tetraploids from Luoning, seven *M. bipartitus* diploids from Daqing and two *P. dabryanus* diploids from Fuyuan (as the outgroup) were employed for cytochrome *b* (Cyt *b*) analysis. Among these specimens, six tetraploids and two diploids of *M. anguillicaudatus*, as well as two diploids of *M. bipartitus* (which were all randomly chosen, but ensuring a 1:1 ratio of sexes in each lineage) and two diploids of *P. dabryanus* were used in further analyses (Table 1).

#### 2.1.2. Amplification, Cloning and Sequencing

Genomic DNA was extracted from preserved fin tissue of each individual loach using a high-salt method and was then employed as a polymerase chain reaction (PCR) template. Cyt *b*, recombination activating gene-1 (RAG-1) and the *V1r1*-*V1r2* cluster were amplified via PCR. For high fidelity, PrimeSTAR Max DNA Polymerase (TaKaRa Biotechnology, Dalian, China) was employed in the amplification reactions. The reaction system contained ten pmol of each primer, 200 ng of genomic DNA and PrimeSTAR Max Premix in a 50-μL reaction volume. The PCR conditions and primers can be found in the Supplementary Materials.

We designed primers to amplify the *V1r1*-*V1r2* clusters containing the complete coding DNA sequences (CDS) of *V1r1* and *V1r2*: Because *V1r1* and *V1r2* exhibit a head-to-head orientation, a partial segment of the *V1r1*-*V1r2* cluster containing the complete intergenic region and partial CDS of *V1r1* and *V1r2* were amplified from a diploid *M. anguillicaudatus* individual, using a pair of primers, one of which corresponded to a V1R2 motif described in a previous study [16] and one of which was designed based on a conserved region of pre-existing V1R1 sequences of model fish species (see the Supplementary Materials), and sequenced on an ABI 3730 capillary sequencer (Applied Biosystems, Foster City, CA, USA). Next, based on the sequence of the partial segment of the *V1r1*-*V1r2* cluster, the flanking regions were obtained using the Genome Walking Kit (TaKaRa Biotechnology) and sequenced. The sequences of the flanking regions were used to design primers for trans-species amplification of the whole *V1r1*-*V1r2* cluster from one *M. bipartitus* individual. A conserved primer pair for the *V1r1*-*V1r2* cluster was then designed based on the conserved flanking regions and was employed for other samples.

Each PCR product was purified and sequenced. When there were sites showing simultaneous signals of double or multiple nucleotides, the purified PCR product was subcloned after the addition of a 3′ adenosine. For each tetraploid sample, at least twenty positive clones (six positive clones for each of the diploid sample) from each RAG-1 and *V1r1*-*V1r2* PCR product were sequenced to ensure at least two representatives of each allele.

### 2.2. Data Analysis

#### 2.2.1. Sequence Characterization and Alignment and Phylogenetic Reconstruction

The identities of the newly-sequenced *V1r* sequences were determined tentatively by the basic local alignment search tool (BLASTX) searches against the RefSeq protein database of *Danio rerio* at the National Center for Biotechnology Information (NCBI) [17]. The orthology of the new *V1r1* and *V1r2* sequences compared with the well-defined *V1r* genes of model fish species was confirmed via phylogenetic analysis employing the neighbor-joining (NJ) method using p-distance in MEGA7 software [18]. The statistical confidence of each node was assessed with 1000 bootstrap replicates. The GenBank accession numbers of newly-obtained cytochrome *b*, RAG-1, *V1r1* and *V1r2* sequences are KX467205–KX467214, KX500017–KX500023, and others are provided in Table 1. Transmembrane regions of the V1R and V2R proteins were predicted by the TMpred server (https://embnet.vital-it.ch/software/TMPRED_form.html) [19].

The obtained sequences of Cyt *b*, RAG-1, *V1r1*, *V1r2* and *V1r1*-*V1r2* cluster were aligned respectively using ClustalW [20] implemented in MEGA7. The model selection program implemented in MEGA7 was used to determine the models of DNA evolution that best fitted our different datasets for phylogenetic tree or network reconstruction. Maximum likelihood (ML) trees were constructed with the selected substitution models by the ML method implemented in MEGA7. The statistical confidence in the nodes was assessed with 1000 bootstrap replicates. The MrBayes 3.1.2 program [21] was used to construct the Bayesian trees with the best available model. Two independent Bayesian analyses were run simultaneously for 10 million generations each sampling every 100th generation. A burn-in of 25,000 trees was removed. The statistical confidence in the nodes of the Bayesian trees was assessed by posterior probabilities. The software SplitsTree4 [22] was used to generate the split networks of the *V1r1*-*V1r2* cluster by the neighbor-net method [23] and ML distances with the best model selected by MEGA7.

#### 2.2.2. Recombination and Selection Analyses

The Φ test [24] was conducted to detect recombination of the *V1r1*-*V1r2* clusters using SplitsTree4. RDP4 software [25] was also employed to detect recombination events in the *V1r1*-*V1r2* cluster of the weather loach tetraploid lineage. Query versus reference recombination scans were adopted using the RDP [26], GENECONV [27], BootScan [28], MaxChi [29], Chimaera [30], SiScan [31] and 3Seq [32] methods implemented in the RDP4 software. The *V1r1*-*V1r2* cluster sequences of the *M. anguillicaudatus* diploids were set as ´Reference Group 1´ and those of *M. bipartitus* as ´Reference Group 2´.

To measure the selection pressure experienced by *V1r1* and *V1r2*, the ratio of nonsynonymous substitutions per nonsynonymous site to synonymous substitutions per synonymous site (d_N_/d_S_) for individual codons of the two genes was calculated using single-likelihood ancestor counting (SLAC) [33], as well as the fixed-effects likelihood method (FEL) [34]. Both methods were employed as implemented by the Datamonkey server [35]. To determine whether the (d_N_/d_S_) ratios differed between the three loach species, the Codeml program of the PAML 4 package [36] was used to estimate likelihood values under the one-ratio and free-ratio models, after which a likelihood ratio test comparing the two models was conducted. The Hudson-Kreitman-Aguadé (HKA) test [37] implemented in DnaSP v5 software [38] was employed to detect selection via pairwise comparisons of RAG-1 and the whole *V1r1*-*V1r2* cluster, RAG-1 and *V1r1* (CDS), RAG-1 and *V1r2* (CDS), and RAG-1 and the intergenic region of the *V1r1*-*V1r2* cluster, based on the polymorphisms in the *M*. *anguillicaudatus* tetraploids and the divergence between the tetraploid lineage and *P*. *dabryanus*.

## 3. Results and Discussion

### 3.1. Identification of an Allotetraploid Lineage of Oriental Weatherfishes

**Figure 1:**
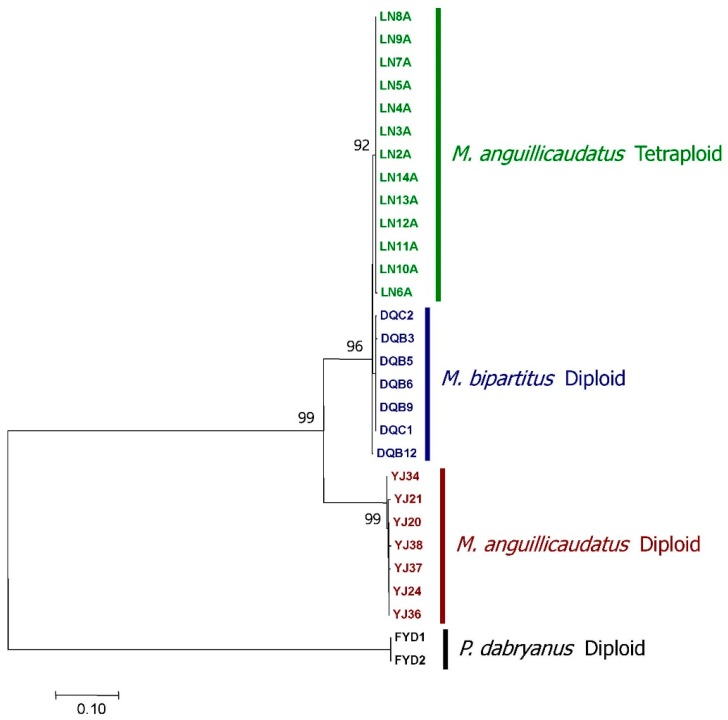
Phylogenetic tree of the diploid and tetraploid *Misgurnus anguillicaudatus*, *M. bipartitus* and *Paramisgurnus. dabryanus* based on cytochrome *b* sequences. The phylogenetic tree was reconstructed by the ML (maximum likelihood) method under the HKY (Hasegawa–Kishino–Yano) + G (gamma distribution) model, as selected by the program in MEGA7. Statistical support values (percentage) for nodes were calculated with 1000 bootstrap replicates in the ML analysis. This phylogenetic tree indicates that *M. bipartitus* was the maternal species of the tetraploid lineage, which was morphologically recognized as *M. anguillicaudatus*. Names for individuals contain abbreviations of the sample locations as in Table 1.

**Figure 2:**
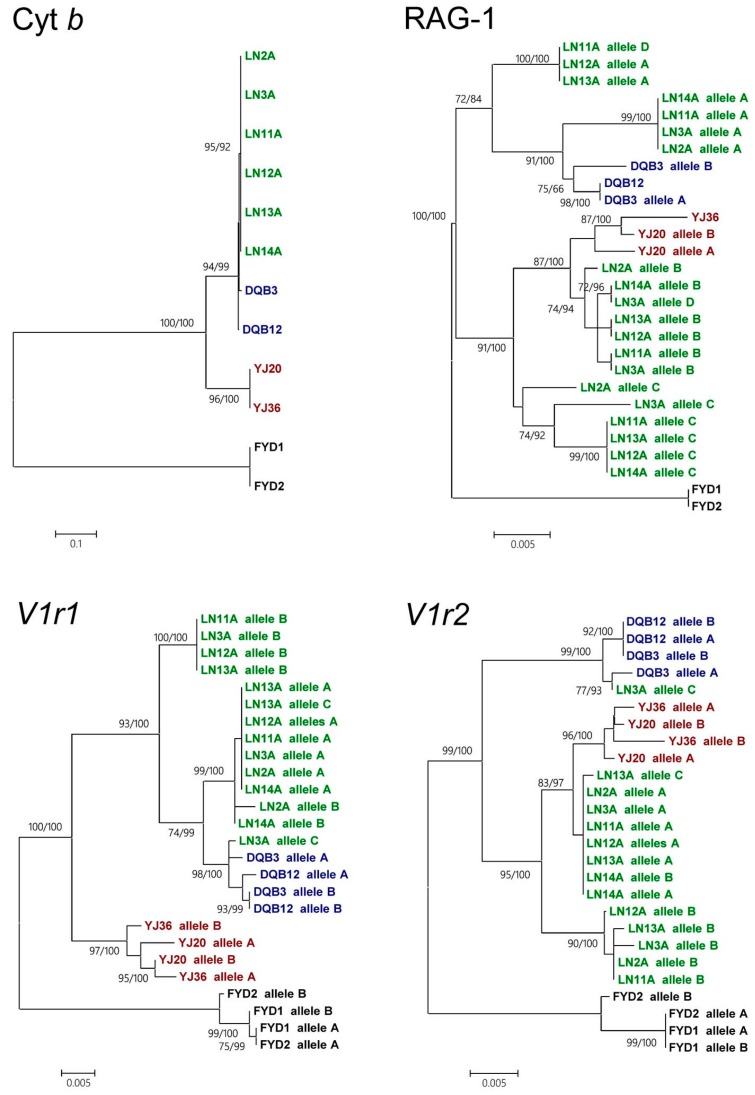
Gene tree comparison among Cyt *b*, RAG-1, *V1r1* and *V1r2* in the same individuals of the diploid and tetraploid *M. anguillicaudatus*, *M. bipartitus* and *P. dabryanus*. ML trees are shown, and the general topologies of Bayesian trees are the same as those of ML trees. Two types of statistical support values for nodes are provided: the first number is the percentage of support calculated with 1000 bootstrap replicates in the ML tree reconstruction, and the second number is posterior probabilities in the Bayesian reconstruction. The substitution models selected for the ML and Bayesian analyses are listed in Table S1. The tree topologies among different genes show incongruence. This incongruence can be explained by the heterogeneity of the genealogy of genes in the genome of tetraploids and thus reveals a hybrid origin of the tetraploid lineage (green) having the ancestral diploid *M. anguillicaudatus* (red) and *M. bipartitus* (blue) as parental species. An alternative explanation for the discordance of gene trees is that it was caused by incomplete lineage sorting, but this explanation is much less plausible since *V1r1* and *V1r2* are tightly linked. Individual names are as in Table 1.

A tetraploid lineage collected in Luoning that was morphologically classified as *M. anguillicaudatus* was identified via the measurement of DNA content using flow cytometry. Individuals of diploid *M. anguillicaudatus*, *M. bipartitus* and *P. dabryanus* were identified in the samples from Yueyang, Daqing and Fuyuan, respectively. A phylogenetic tree that was constructed using cytochrome *b* sequences (1140 bp, complete CDS) and received high statistical support indicated that the tetraploid individuals were clustered together and nested within *M. bipartitus*. Therefore, the maternal ancestor of the tetraploid lineage was *M. bipartitus* and not *M. anguillicaudatus*, in contrast to the observed morphological resemblance (Figure 1), suggesting an allotetraploid origin of the tetraploid lineage. Because of the extremely low haplotype diversity of Cyt *b* observed in the tetraploid lineage and the nesting of Cyt *b* within *M*. *bipartitus*, it could be inferred that the formation of the tetraploid lineage was quite recent. Moreover, the tree constructed based on RAG-1 alleles (903 bp, partial CDS) showed that each tetraploid individual exhibited three to four RAG-1 alleles, among which at least one clustered with diploid *M. anguillicaudatus* and at least one clustered with *M. bipartitus* (Figure 2). This tree topology indicated that the RAG-1 alleles of the tetraploid lineage likely originated from both of the diploid *M. anguillicaudatus* and *M. bipartitus*, which means that in the genome of the tetraploid lineage, the RAG-1 alleles originated from the diploid *M. anguillicaudatus* and the RAG-1 alleles originated from *M. bipartitus* are homoeologs, which originated by speciation of parental species and were brought together in one genome by allopolyploidization [39]. If the RAG-1 homoeologs are true alleles at one locus with polysomic inheritance, then it is expected that one homoeolog will be absent in a certain proportion of tetraploid individuals. Because every tetraploid individual possesses both RAG-1 homoeologs in our analysis, the homoeologs actually behave as paralogs at two loci of the tetraploid genome with disomic inheritance. The morphological resemblance to the diploid *M*. *anguillicaudatus*, the nesting of Cyt *b* within *M*. *bipartitus* and the preservation of alleles descended from both parental species confirm an allotetraploid origin of the tetraploid lineage.

### 3.2. Characterization of the V1r1 and V1r2 Genes of Weatherfishes

One pair of *V1r1* and *V1r2* was identified based on the sequenced segments of the *V1r1*-*V1r2* clusters of *M. anguillicaudatus*, *M. bipartitus* and *P. dabryanus*. The identities of individual *V1r* genes were first checked using BLASTX against the RefSeq protein database of *Danio rerio* and were then confirmed via phylogenetic reconstruction using the V1R1 and V1R2 sequences of model teleost fishes (Figure S2). The complete CDS of *V1r1* and *V1r2* encoded 322 and 317 amino acids, respectively, in all three weatherfishes and were longer than those of zebrafish (303 and 316 amino acids, respectively [4]). In all three loach species, *V1r1* and *V1r2* were arranged in a head-to-head manner, which is consistent with other teleost fishes [6]. The *V1r1* and *V1r2* intergenic region was approximately 1.5 kb in all three species, which is shorter than those of other surveyed teleosts [6].

### 3.3. Recombination between Homoeologous V1r1-V1r2 Clusters in the Tetraploid Genome

When the *V1r1* and *V1r2* sequences of tetraploid *M. anguillicaudatus* and diploid *M. anguillicaudatus*, *M. bipartitus* and *P. dabryanus* were used to construct phylogenetic trees, there was discordance of tree topologies between *V1r1* and *V1r2* (Figure 2). In the *V1r2* tree, all *V1r2* sequences of tetraploid *M. anguillicaudatus* except for one clustered with those of diploid *M. anguillicaudatus*, suggesting that the *V1r2* of tetraploid *M. anguillicaudatus* descended from diploid *M. anguillicaudatus*. However, all of the tetraploid *M. anguillicaudatus V1r1* sequences clustered with *M. bipartitus* sequences in the *V1r1* tree, suggesting that the *V1r1* of tetraploid *M. anguillicaudatus* descended from *M. bipartitus*, rather than from diploid *M. anguillicaudatus*. The combined pattern of the *V1r1* and *V1r2* trees with different genealogies suggested that the tetraploid lineage possessed a recombinant *V1r1*-*V1r2* cluster, in which *V1r1* was derived from *M. bipartitus* and *V1r2* was derived from diploid *M. anguillicaudatus*. Discordance of tree topologies between different genes can result from either introgressive hybridization or incomplete lineage sorting [40]. Incomplete lineage sorting, as an alternative explanation for the incongruence of gene tree topologies, is implausible because *V1r1* and *V1r2* are tightly linked.

**Figure 3:**
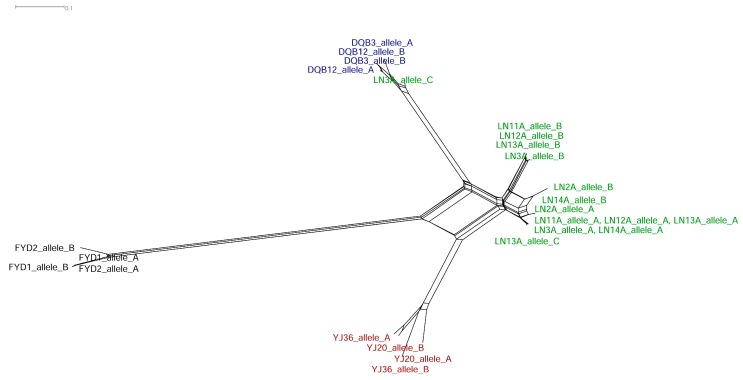
Split network of the *V1r1*-*V1r2* clusters in the weatherfishes containing complete coding DNA sequences (CDS) of *V1r1* and *V1r2*, and the intergenic region between them. The split network was constructed based on ML distances under the selected best model K2 (Kimura two-parameter) + G and clearly visualizes the hybridization signal, which shows the hybrid nature of the *V1r1*-*V1r2* clusters (with one exception) in the tetraploid lineage. Individual names are as in Table 1. Tetraploid lineage (green), diploid *M. anguillicaudatus* (red), *M. bipartitus* (blue), *P. dabryanus* (black).

Using *V1r1*-*V1r2* cluster sequences (provided in the Supplementary Materials) containing the complete coding region and the approximately 1.5-kb intergenic region, we further tested the hypothesis of hybridization and recombination by split-network reconstruction and several recombination detection methods. Signals of recombination can be visualized via split-network reconstruction with the potential parental and recombinant sequences [22]. As expected, the constructed split-network based on the full-length *V1r1*-*V1r2* sequences showed recombination in allotetraploid individuals (Figure 3). The only exception was an allele from one tetraploid individual (LN3A allele C) that was derived from *M*. *bipartitus* and was of non-recombinant origin.

**Figure 4:**
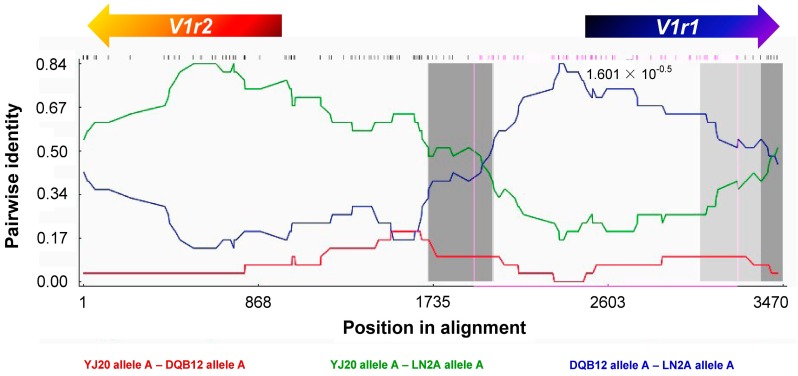
An example of the recombination test of *V1r1*-*V1r2* clusters by sliding window analysis using the RDP method. On the horizontal axis is the site position of DNA alignment; on the vertical axis is the pairwise identity of a 30-bp window sliding along the sequences. YJ20, DQB12 and LN2A are individual names as in Table 1. The direction of the clusters is *V1r2* (in reverse)-intergenic region-*V1r1*. This figure shows that in the first part (containing *V1r2*) of the cluster, the identity between LN2A (tetraploid lineage) and YJ20 (diploid *M. anguillicaudatus*) is much higher than that between LN2A and DQB12 (*M. bipartitus*); in contrast, in the latter part (containing *V1r1*) of the cluster, the identity between LN2A and DQB12 is much higher than that between LN2A and YJ20. The arrows on the top show the positions and directions of CDS regions of *V1r1* and *V1r2*, respectively. The small lines below the arrows mark the variable sites. This analysis indicates that the *V1r1*-*V1r2* cluster of the tetraploid LN2A is a recombinant from that of diploid *M. anguillicaudatus* and *M. bipartitus* and that the breakpoint position was near the middle of the intergenic region. Similar results were acquired in the analyses of the other sequences of tetraploids except one (LN3A allele C).

The evidence of recombination provided by the Φ test was statistically significant (*P* = 5.827 × 10^−6^ < 0.001). When the breakpoints and statistical significance of the *V1r1*-*V1r2* recombination events were estimated via the RDP, GENECONV, BootScan, MaxChi, Chimaera, SiScan and 3Seq methods, recombination events were detected with statistical support in all sequences except for one (see the Supplementary Materials). Consistent with the *V1r1* and *V1r2* tree topologies, the recombination breakpoint lies between the *V1r1* and *V1r2* intergenic region and partitions the cluster such that the *V1r1* region is derived from *M. bipartitus* and the *V1r2* region is derived from *M. anguillicaudatus* (Figure 4).

**Figure 5:**
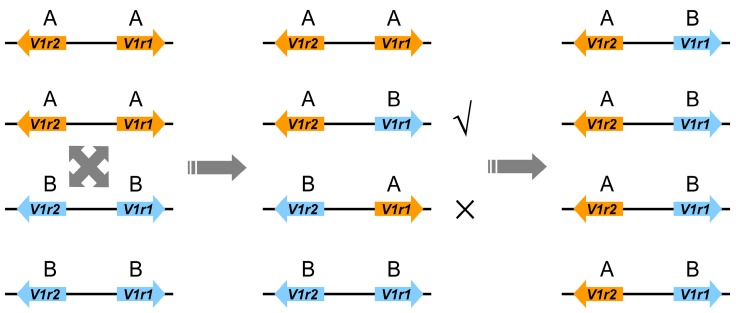
A proposed evolutionary model of the *V1r1*-*V1r2* cluster in the allotetraploid loach genome. ´A´ denotes deriving from diploid *M. anguillicaudatus*, and ´B´ denotes deriving from *M. bipartitus*. Initially, the A-A type of the *V1r1*-*V1r2* cluster and the B-B type coexisted in the genome of the allotetraploid individuals, then intergenic recombination between homoeologs happened, and the recombinant A (*V1r2*)-B (*V1r1*) type, which probably possesses an evolutionary advantage, progressed toward fixation with polysomic inheritance and now almost gets fixed in the tetraploid lineage. The B (*V1r2*)-A (*V1r1*) type, the other type of recombinant, was excluded in the descendants of early tetraploids perhaps due to being less favorable than the A (*V1r2*)-B (*V1r1*) type or even being selected against.

Accordingly, we propose a model describing the recombination of the parental *V1r1*-*V1r2* cluster and the differential preservation of recombinant forms (Figure 5). In this model, the parental *V1r1*-*V1r2* clusters coexisted in the genomes of the allotetraploid population at the beginning, then intergenic recombination between homoeologs generated two recombinant types. The recombinant type harboring *V1r2* from *M*. *anguillicaudatus* and *V1r1* from *M*. *bipartitus* persists and is predominant today in the tetraploid lineage, while the other recombinant type and parental types were excluded or rare in the descendants of early tetraploids.

The recombinant *V1r1*-*V1r2* cluster in which *V1r1* is derived from *M. bipartitus* and *V1r2* from the diploid *M. anguillicaudatus* is dominant in the present tetraploid population. This pattern suggests that this recombinant has been selected for over the parental types and the other recombinant type and that there has been selection against the heterogeneity of *V1r* alleles, namely the coexistence of the parental *V1r1*s and *V1r2*s, considering that the parental *V1r1*-*V1r2* clusters should coexist in the allotetraploid population in the early stage and homoeologous recombination would produce two types of recombinants with an equal frequency. If there were no positive selection on the recombinant, it would have little chance to persist in the population, let alone become dominant or even fixed, because the initial frequency of the recombinant must be far lower than that of the parental types since the recombination event at a certain position is relatively rare. However, actually, the recombinant is currently dominant and near fixation. If there were no purifying selection against the heterogeneity of *V1r* alleles, the favored combination, for which *V1r1* derived from *M. bipartitus* and *V1r2* derived from the diploid *M. anguillicaudatus*, could be achieved by the coexistence of parental types of the *V1r1-V1r2* cluster. Then, the recombinant *V1r1-V1r2* cluster was not necessarily needed, which would make it hard for the recombinant to be dominant even if the type of combination of *V1r1* and *V1r2* it afforded was favored. Meanwhile, the origin of the tetraploid lineage is recent, which is suggested by the low haplotype diversity and the nesting of the tetraploids within *M. bipartitus* in the Cyt *b* tree (Figure 1). Hence, the selective force must be strong enough for the near-complete fixation of the recombinant *V1r1*-*V1r2* cluster, which occurred within a short time period.

One of the possible reasons why the recombinant *V1r1*-*V1r2* cluster has a selective advantage is the following: new combinations of V1Rs could contribute to detecting new pheromones in two levels. First, for deciphering the information of a single chemical compound, a combination of different V1R receptors is required to couple molecules of this compound to transmit its signal [41,42]. Second, a pheromone may comprise multiple compounds, which demands higher complexity of receptor combination to fully detect and transmit the signal of the pheromone [43]. Hence, the new *V1r1*-*V1r2* combination identified in the tetraploid loach (with *V1r1* from *M. bipartitus* and *V1r2* from diploid *M. anguillicaudatus*) has possibly imparted an ability to detect a lineage-specific pheromone, which might have a hybrid nature due to the hybrid origin of the tetraploid lineage.

### 3.4. Divergence of V1r1 and V1r2 among the Diploid Parental Species and the Tetraploids

We measured selective pressure to identify sites under positive selection in the *V1r1* and *V1r2* sequences of diploid *M. anguillicaudatus*, *M. bipartitus* and *P. dabryanus* by calculating the d_N_/d_S_ ratios for individual codons using the SLAC and FEL methods. In general, the d_N_/d_S_ ratios were far less than one, indicating that the codons were under purifying selection. No positively selected sites were identified in the above analyses. The selective pressures measured using the *V1r1* d_N_/d_S_ ratios were not significantly different among the three diploid species according to the likelihood ratio test (which compares the one-ratio and free-ratio models) by PAML. This pattern also applied to *V1r2*. 

**Figure 6:**
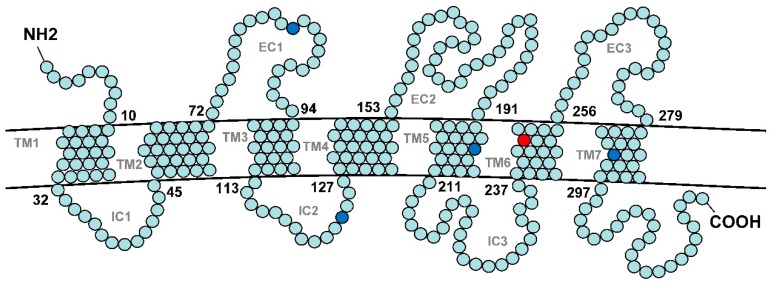
The predicted protein structure of V1R1 of the tetraploid weatherfish. The predicated protein structure based on the sequence of *V1r1* allele A of the tetraploid individual LN12A is shown as a representative. The predicted V1R1 structures are alike among the diploid and tetraploid *M. anguillicaudatus* and *M. bipartitus*. The amino acid sites that are fixed in the tetraploid *M. anguillicaudatus* and are the same as those for *M. bipartitus*, but distinct from those of the diploid *M. anguillicaudatus*, are highlighted in blue. The amino acid site that is fixed in the tetraploid *M. anguillicaudatus* and is the same as that of the diploid *M. anguillicaudatus*, but distinct from that of *M. bipartitus*, is highlighted in red. Note that point mutations of transmembrane region (TM) 5 and TM6 can change the ligand-binding specificity of an olfactory receptor [44]. IC: intracellular; EC: extracellular.

By comparing the predicted V1R protein sequences, we found that the *V1r1* proteins of *M. bipartitus* were distinct from those of diploid *M. anguillicaudatus* at eight fixed sites (17-S/A, 78-N/D, 84-V/A, 124-V/L, 200-I/M, 251-V/L, 289-I/V, 311-P/L), two of which (200, 251) were within the putative key regions (Transmembrane region (TM) 3, TM5 and TM6) that impact ligand-binding specificity of an olfactory receptor [44]. The *M. bipartitus* residues in four of the fixed sites (84-V, 124-V, 200-I, 289-I) were inherited by the tetraploid population. One (200-I) occurred in the TM5 key region (Figure 6). However, in all of the recombinant *V1r1*-*V1r2* clusters of tetraploids, the residue at position 251, located in the TM6 key region of *V1r1*, was the same as that of diploid *M*. *anguillicaudatus*, rather than *M*. *bipartitus* (L instead of V). This recombination likely allowed *V1r1* to evolve a novel ligand-binding specificity. In contrast to *V1r1*, none of the amino acid residues of *V1r2* that were differentially fixed between diploid *M. anguillicaudatus* and *M. bipartitus* were located in the putative key functional regions, and none of these residues were fixed in the tetraploid population.

### 3.5. Heterogeneous Evolution between Nuclear Genes

The neutral theory of evolution predicts that the intraspecific polymorphism and interspecific divergence will be both high in a gene exhibiting a high mutation rate, while the intraspecific polymorphism and interspecific divergence will be both low in a gene exhibiting a low mutation rate. When comparing two unlinked genes, it can be tested whether the intraspecific polymorphism and interspecific divergence are consistent between the two genes. In the HKA test, expected values of intraspecific polymorphism and interspecific divergence are inferred from the observed data using the neutral theory, and a Χ^2^ statistic is constructed to test whether the observed data fit the neutral expected values [37].

Under the assumption of tetrasomic inheritance in the tetraploid lineage of *M*. *anguillicaudatus*, HKA analysis comparing the polymorphisms within the tetraploid lineage and the divergence between the tetraploid lineage and *P. dabryanus* at the whole *V1r1*-*V1r2* cluster and RAG-1 loci indicated that the null hypothesis of neutral evolution was refused at *P* < 0.1 level, which implicated that the selective pressure acting on the *V1r1*-*V1r2* cluster as a whole was different from that on RAG-1 within the allotetraploid lineage (Table S2). In this comparison, the observed value of the intraspecific polymorphism of the *V1r1-V1r2* cluster is lower than the expected value; in contrast, the observed value of the intraspecific polymorphism of RAG-1 is higher than the expected one. Meanwhile, the observed value of the interspecific divergence of the *V1r1-V1r2* cluster is higher than the expected value; in contrast, the observed value of the interspecific divergence of RAG-1 is lower than the expected one. This pattern can be explained by that the whole *V1r1-V1r2* cluster likely having experienced directional selection, which would increase the interspecific divergence and/or reduce the intraspecific polymorphism. Alternatively, this can be explained by that RAG-1 likely experienced balancing selection, which would increase the intraspecific polymorphism. These two explanations are not mutually exclusive. The *V1r1* and RAG-1 loci also showed a heterogeneous pattern of evolution in the allotetraploid lineage (*P* < 0.1), but such heterogeneity is not obvious between the *V1r2* and RAG-1 loci (Table S2). Interestingly, this pattern of heterogeneous evolution also applied to the *V1r1*-*V1r2* intergenic region and RAG-1 in the allotetraploid lineage with even stronger statistical support (*P* < 0.001) (Table S2). This pattern, in which the *V1r1*-*V1r2* intergenic region has experienced more intense directional selection than RAG-1 and even *V1r1* and *V1r2* raises the possibility that the recombinant intergenic region, which contains the 5′ untranslated regions (UTRs) of *V1r1* and *V1r2*, is under stricter functional constraints than the CDS regions themselves. Thus, directional selection might have primarily acted on the recombined intergenic region, and the decline in *V1r1* and *V1r2* CDS polymorphism is thus more likely caused by the hitchhiking effect rather than by positive selection on the CDS regions *per se*. Such a hitchhiking event could result from the possible situation that in the allotetraploid lineage, the members of translation-associated proteins that interact with the 5’ UTRs of *V1r1* and *V1r2* have a selected hybrid type of combination; in other words, they are derived from different parental species, and the combination pattern is selected for [45].

## 4. Conclusions

We investigated the evolutionary pattern of the sex pheromone receptor gene cluster *V1r1*-*V1r2* in a tetraploid lineage of weather loach. This lineage originated from hybridization between diploid *M. anguillicaudatus* and *M. bipartitus*. The tetraploid lineage harbors a recombinant *V1r1*-*V1r2* cluster in which *V1r1* is derived from *M. bipartitus* and *V1r2* is derived from diploid *M. anguillicaudatus*. This recombinant is almost fixed in the tetraploid population. Selection analyses suggested that there are both positive selection for a specific combination of *V1r* paralogs and purifying selection for homogeneity in each gene, and the *V1r1*-*V1r2* cluster is likely under more intense directional selection than RAG-1. This pattern of *V1r1*-*V1r2* evolution in the recently-formed tetraploid lineage provides an example for how the number of *V1r1* and *V1r2* genes in a teleost genome will regress to one for each after genome duplication.

## Figures and Tables

**Table 1:** Information for the specimen and sequence accession number.

Species	Sample Number	Sex	Location	Accession Number			
				Cyt *b*	RAG-1	*V1r1*	*V1r2*
*Misgurnus anguillicaudatus* (tetraploid race)	LN2A	♀	Luoning, Henan	KX189347	KX189359, KX189360, KX189361	KX189377, KX189378	KX189413, KX189414
	LN3A	♀	Luoning, Henan	KX189348	KX189362, KX189363, KX189364, KX189365	KX189379, KX189380, KX189381	KX189415, KX189416, KX189417
	LN11A	♂	Luoning, Henan	KX189349	KX189366, KX189367, KX189368, KX189369	KX189392, KX189393	KX189418, KX189419
	LN12A	♂	Luoning, Henan	KX189350	KX189370, KX189371, KX189372	KX189394, KX189395	KX189420, KX189421
	LN13A	♀	Luoning, Henan	KX189351	KX189373, KX189374, KX189375	KX189396, KX189397, KX189398	KX189422, KX189423, KX189424
	LN14A	♂	Luoning, Henan	KX189352	KX189376, KX189377, KX189378	KX189399, KX189400	KX189425, KX189426
*Misgurnus anguillicaudatus* (diploid race)	YJ20	♂	Yueyang, Hunan	KX189353	KX189379, KX189380	KX189401, KX189402	KX189427, KX189428
	YJ36	♀	Yueyang, Hunan	KX189354	KX189381	KX189403, KX189404	KX189429, KX189430
*Misgurnus bipartitus* (diploid)	DQB3	♀	Daqing, Helongjiang	KX189355	KX189382, KX189383	KX189405, KX189406	KX189431, KX189432
	DQB12	♂	Daqing, Heilongjiang	KX189356	KX189384	KX189407, KX189408	KX189433, KX189434
*Paramisgurnus dabryanus* (diploid)	FYD1	♀	Fuyuan, Heilongjiang	KX189357	KX189385	KX189409, KX189410	KX189435, KX189436
	FYD2	♂	Fuyuan, Heilongjiang	KX189358	KX189386	KX189411, KX189412	KX189437, KX189438

## Supplementary Materials

The following are available online at www.mdpi.com/2073-4425/8/11/334/s1. Figure S1: Sampling sites on a map, Figure S2: Unrooted phylogenetic tree of V1R1 and V1R2 proteins of *M. anguillicaudatus* (diploid), *M. bipartitus*, *Paramisgurnus dabryanus* and three model fishes, Table S1: Models for phylogenetic construction of different sets of sequences, Table S2: Results of HKA test.
