# Supplementary material for: Homoeologous Recombination of the V1r1-V1r2 Gene Cluster of Pheromone Receptors in an Allotetraploid Lineage of Teleosts
Source: Genes (Basel). 2017 Nov 21;8(11):334. doi: 10.3390/genes8110334 (PMC5704247; doi:10.3390/genes8110334)
Supplement: Supplementary file 1 [file genes-08-00334-s001.zip › Supplementary Materials.pdf]

Supplementary Materials

1. PCR conditions:

1.1. For cytochrome *b*:

After one step of 95 °C for 5 min, 35 successive cycles of 94 °C for 30 sec, 54 °C for 30 sec, and 72 °C for 45 sec.

1.2. For *RAG1*:

After one step of 95°C for 5 min, 30 successive cycles of 94 °C for 45 sec, 49 °C for 45 sec, and 72 °C for 1 min.

1.3. For *V1r1-V1r2* cluster:

After one step of 95°C for 5 min, 30 successive cycles of 94 °C for 45 sec, 48 °C for 45 sec, and 72 °C for 3 min.

2. PCR primer pairs:

2.1. For cytochrome *b*

cytb-F: GACTTGAAGAACCACCGTTGTTATTCAAC

cytb-R: GCGCTAGGGAGGAATTTAACCTCC

2.2. For *RAG1* [1]

RAG-1F: AGCTGTAGTCAGTAYCACAARATG

RAG-RV1: TCCTGRAAGATYTTGTAGAA

2.3. For a partial segment of *V1r1-V1r2* cluster:

aV1r1R: GTBAGVGTRTABACCCAVAGBCC (designed based on pre-existing *V1r1* sequences of *Danio rerio*, *Gasterosteus aculeatus* and *Oryzias latipes*)

aV1R2R: CGCTTTCACCTTCCTGTTGGAGGAGATGAT [2]

2.4. For *V1r1*-*V1r2* cluster (containing complete CDSs of *V1r1* and *V1r2*)

fV1r1: GTGTTTTAGTTCAAGATAAGCTGTG

fV1r2: CCCAATTACAACTCTTTCCTGAG

[1] Slechtova, V., Bohlen, J. and Perdices, A. (2008). Molecular phylogeny of the freshwater fish family Cobitidae (Cypriniformes : Teleostei): Delimitation of genera, mitochondrial introgression and evolution of sexual dimorphism. Molecular Phylogenetics And Evolution 47, 812-831.

[2] Pfister, P. and Rodriguez, I. (2005). Olfactory expression of a single and highly variable V1r pheromone receptor-like gene in fish species. Proceedings Of the National Academy Of Sciences Of the United States Of America 102, 5489-5494.

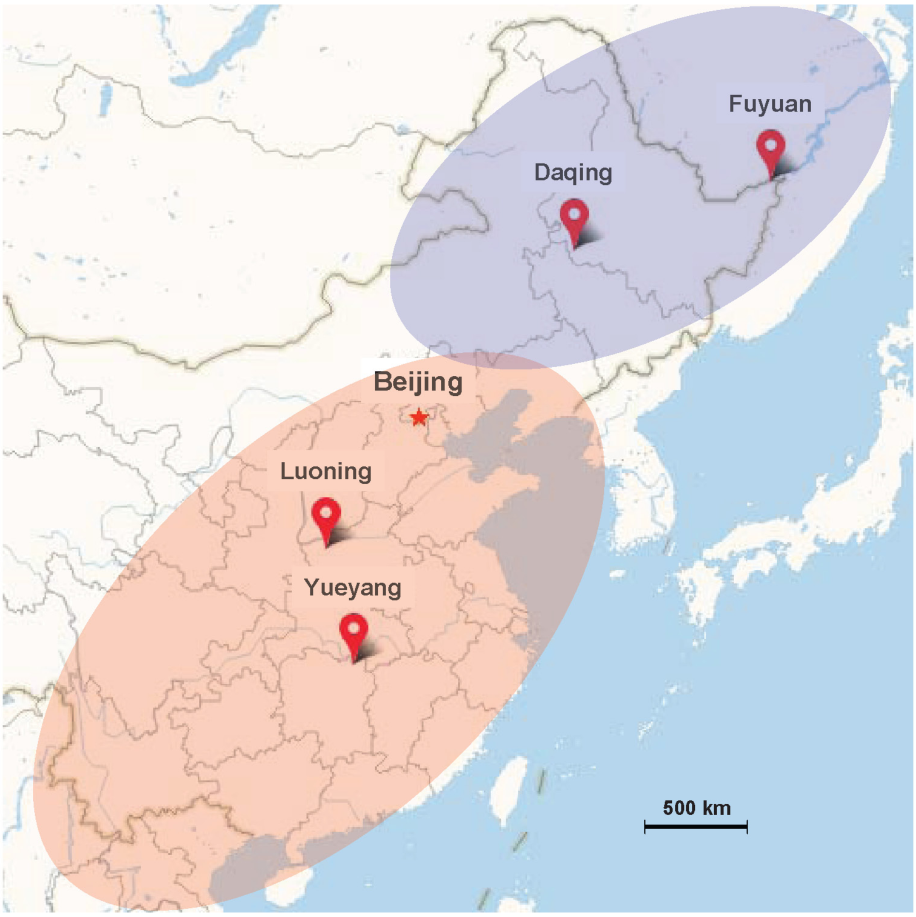

Figure S1 – Sampling sites on a map.

The general distribution range of *Misgurnus anguillicaudatus* is shaded in orange and that of *M. bipartitus* in purple, by simplification. Populations of *Paramisgurnus dabryanus* live in all these areas. In this study, diploids of *M. anguillicaudatus* were collected in Yueyang, tetraploids of *M. anguillicaudatus* in Luoning, samples of *M. bipartitus* in Daqing and samples of *Paramisgurnus dabryanus* in Fuyuan.

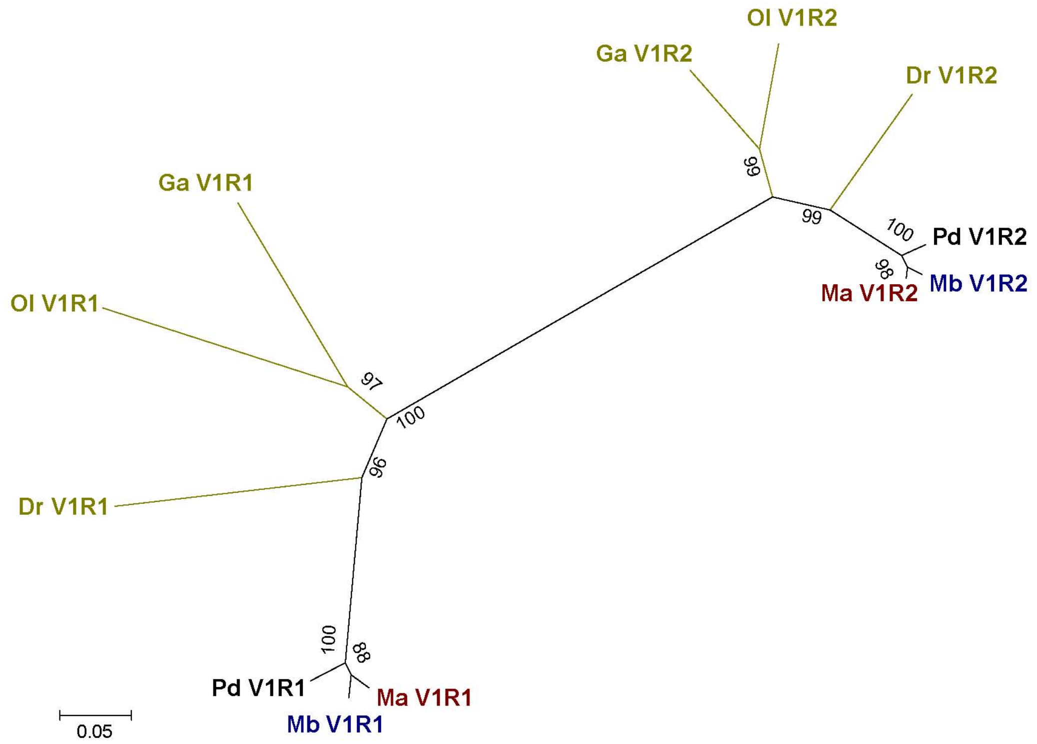

Figure S2 – Unrooted phylogenetic tree of V1R1 and V1R2 protein of *M. anguillicaudatus* (diploid), *M. bipartitus*, *Paramisgurnus dabryanus* and three model fishes.

This phylogenetic tree was constructed by Neighbour-Joining (NJ) method using p-distance. Statistical support values (percentage) for nodes were calculated with 1000 bootstrap replicates. Abbreviations: Dr, *Danio rerio*; Ga, *Gasterosteus aculeatus*; Ol, *Oryzias latipes*; Ma, *M. anguillicaudatus*; Mb, *M. bipartitus*; Pb, *P. dabryanus*.

Table S1 – Models for phylogenetic construction of different sets of sequences.

Gene	Model selected by MEGA7	Model used in ML	Model used in MrBayes
Cyt *b*	HKY+G	HKY+G	HKY+G (nst=2 rates=gamma)
RAG1	K2+G	K2+G	HKY+G (nst=2 rates=gamma)
V1r1	K2+G	K2+G	HKY+G (nst=2 rates=gamma)
V1r2	T92+G	T92+G	HKY+G (nst=2 rates=gamma)

Abbreviations: HKY: Hasegawa-Kishino-Yano; T92: Tamura 3-parameter; K2: Kimura 2-parameter.

Table S2 – Results of HKA test.

	Comparison 1		Comparison 2		Comparison 3		Comparison 4	
	RAG-1	*V1r1-V1r2* cluster	RAG-1	*V1r1*	RAG-1	*V1r2*	RAG-1	Intergenic region
**Intraspecific Ploymorphism**								
Segregating Sites (observation)	42	97	42	24	42	28	42	14
Segregating Sites (expectation)	28.71	110.29	32.20	33.80	37.01	32.99	23.26	32.74
Total Number of Sites	903	3361	903	969	903	954	903	1412
Sample Size	24	24	24	24	24	24	24	24
**Interspecific Divergence**								
No. Differences (observation)	26.75	167.11	26.75	48.18	26.75	33.29	26.75	82.75
No. Difference (expectation)	40.04	153.82	36.55	38.38	31.74	28.29	45.49	64.01
Total Number of Sites	903	3361	903	969	903	954	903	1412
chi-square test Value	3.502		2.883		0.712		13.018	
P-value	0.0613		0.0895		0.3989		0.0003	

Alignment of the *V1r1-V1r2* clusters of samples is provided as an independent file.

Summary of the recombination analysis by RDP4 is provided as an independent file.
